# Recent Advances of Stem Cell Therapy for Retinitis Pigmentosa

**DOI:** 10.3390/ijms150814456

**Published:** 2014-08-20

**Authors:** Yuxi He, Yan Zhang, Xin Liu, Emma Ghazaryan, Ying Li, Jianan Xie, Guanfang Su

**Affiliations:** Department of Ophthalmology, Second Hospital, Jilin University, Ziqiang Street No. 218, Changchun 130041, Jilin, China; E-Mails: heyuxihot@163.com (Y.H.); zhangy66@jlu.edu.cn (Y.Z.); emperorsoninlow@163.com (X.L.); emmaghazaryan@163.com (E.G.); lyvoilet@163.com (Y.L.); 13844108986@163.com (J.X.)

**Keywords:** retinitis pigmentosa, bone marrow-derived mesenchymal stem cells, stem cell transplantation

## Abstract

Retinitis pigmentosa (RP) is a group of inherited retinal disorders characterized by progressive loss of photoreceptors and eventually leads to retina degeneration and atrophy. Until now, the exact pathogenesis and etiology of this disease has not been clear, and many approaches for RP therapies have been carried out in animals and in clinical trials. In recent years, stem cell transplantation-based attempts made some progress, especially the transplantation of bone marrow-derived mesenchymal stem cells (BMSCs). This review will provide an overview of stem cell-based treatment of RP and its main problems, to provide evidence for the safety and feasibility for further clinical treatment.

## 1. Introduction

Retinitis pigmentosa (RP) is a group of inherited retinal disorders characterized by progressive loss of photoreceptors and eventually leads to retina degeneration and atrophy. New approaches for RP therapies include: cell transplantation therapy [1], gene therapy [2,3], cytokine therapy [4], nutrition therapy [5,6,7], and hyperbaric oxygen therapy [8]. Present therapies for RP are restricted in their efficacy or safety, for example: maintainence of long-term efficacy using a single injection of cytokines is difficult, but there is a risk of infection after repeated intra-vitreous injections in cytokine therapy [9,10]. Gene therapy has been shown to improve visual function in inherited retinal disease [11,12]. RP is a hereditary cause of blindness, which has four main modes of inheritance: Autosomal dominant RP (ADRP), autosomal recessive RP (ARRP), X-linked RP (XLRP) and dihybrid inheritance, also mitochondrial genetic and non-genetic forms [13,14,15,16]. Thomas and colleagues injected *rAAV2-VMD2-hMERTK* vector into the subretinal space of RCS rats and SD rats; it showed improvement of visual function in RCS, they also performed a series of safety studies in normal SD rats, and demonstrated that no local or systemic toxicity was detected after either dose of vector delivery and no indication of vector spread outside the treated eye [17]. This group also prompted a phase I clinical trial in which *rAAV2-VMD2-hMERTK* vector were injected into the subretinal space of patients with retinal disease due to *MERTK* mutations.

In recent years, cell transplantation therapy in retinitis pigmentosa has made progress. Researchers have found that the transplanted cells can be used as a substitute for degenerated cells or they can release some growth factors to enhance cell survival, growth and function of retinal cells. Transplanted cells types are: retinal pigment epithelium (RPE) [1], schwann cells [18], central nervous system stem cells [19], olfactory ensheathing cells [20], retinal stem cells [21], hematopoietic stem cells [22] and embryonic stem cells [23]. However, the clinical use of these cells have several problems, such as: limited availability of pluripotent retinal stem cells [24], and rejection by the host immune system in either allogeneic or xenogenic host species recipients [25], thus researchers prefer BMSCs for transplantation therapy. Table 1 shows a comparison of other source of stem cells used for stem cell therapy.

**Table 1:** Comparison of other source of stem cells used for stem cell therapy in degenerative retinopathies disease.

Type of Stem Cells	Origin	Advantage	Disadvantage
**Retinal progenitor cells**	Derived from fetal or neonatal retinas [21], if isolated from the developing retina at a suitable stage, photoreceptor precursors may be obtained [26].	Can migrate into retinal layers, develop morphological characteristics of various retinal cell types [27].	Ethical and immune rejection issues [28].
**Embryonic stem cells (ESC)**	Derived from inner cell mass of blastocyst-stage embryos.	ESC can differentiate into photoreceptor progenitors, photoreceptor, or retinal pigment epithelium (RPE) [29,30,31].	Ethical and immune rejection issues, associated with teratoma formation [32,33].
**Induced Pluripotent Stem Cells (iPS)**	Pluripotent ESC-like cells reprogrammed * in vitro* from terminally differentiated somatic cell [34].	Use as disease model by integrating iPS derived from retinitis pigmentosa patient [35]. iPS can differentiate into functional RPE [35] and photoreceptor precursor cells [36,37].	It has the risk of viral integrations and oncogene expression [38].
**Mesenchymal Stem Cells (MSC)**	Bone marrow derived cell population, have the ability to self-renew and give rise to multiple tissue types [39]. Other sources of MSC including adipose tissue, placenta and cord blood [40,41,42,43].	Could be induced into cells expressing photoreceptor markers [44], the experiment demonstrated that the cells slow down retinal cell degeneration [45]. Some even carried on clinical trials [46,47,48].	Low rate of cell survival and migration in the retina [49]. Biosafety issues.
**Olfactory Ensheathing Cells (OECs)**	A type of glia cells capable of continuous growth and regeneration of olfactory axons into the CNS [50,51].	Cleaned up the accumulated debris in subretinal space, and provided an intrinsic continuous supply of neurotrophic factors, reduced the gliotic injury response of Muller cells [20,52].	Mainly used to promote regeneration of lesion spinal cord axons [50].
**Human Neural Progenitors**	In the CNS, the cells derived from prenatal cortex.	Could rescue long-term vision function and associated morphologic substrates in a rat model of photoreceptor degeneration [53,54].	Protected dying host neurons within both the brain and spinal cord [55,56].

## 2. The Problems Exist in Gene Therapy 

Most gene therapies involved integration of vector DNA into the specific cells of retina [17,57], and even combine RNA interference (RNAi)-based gene silencing with gene replacement in RP [58,59]. Gene therapy needs to comply with the following principles: first, the genetic material of the pathogenic cells which suffered deletions or mutations of genes should be modified to recover part or all of functions, and second, the expression of genetic material should have no side effects in specific cells. Viruses are widely used as vectors for gene therapy and they are small in size and can transduce many cell types.

However, several problems still exist in gene therapy: (1) Gene therapy to introduce trophic factors or to correct mutated genes may help in the early stages [60,61,62], but this treatment is less effective with disease progression. Only recently, attempts at using AAV2/8 gene therapy to functionally rescue *Pde6β* mouse models of RP, has shown long-term rescue effects [12,63]. It was not found when using lentiviral, or other AAV serotypes in the majority of the reported studies [61,62]. And only one AAV gene therapy case has now shown a long-term rescue in a slow photoreceptor degeneration *Pde6β* mouse model; studies have not been conducted on other kind of animal models of RP; (2) Despite the typical hereditary RP, there are still many reports about the lack of retinal disease with a family history, these patients are called—Sporadic retinitis pigmentosa (SRP). The majority of the SRP are confirmed as ARRP [64], and some others are dihybrid inheritance [65] and mitochondrial genetic forms [66]. Especially the onset time was adult and elderly in patients, this type of RP may be non-hereditary [16], gene therapy has no effect on this kind of RP; (3) An experiment also demonstrated the treatment using AAV-mediated did not significantly ameliorate cell death. Schlichtenbrede and his colleagues proved that significant loss of retinal function after intraocular gene delivery of ciliary neurotrophic factor with AAV, both in normal mice and in the model of RP [67]; (4) Viruses are widely used, but they have limitations, gene expression decreased over time [68], and manufactured less cost effectively. However, the efficiency and duration of ordinary nonviral vector-mediated gene transfer to the retina are rather poor compared with viral vectors. Delgado and colleagues report that they used rational nonviral vectors based on solid lipid nanoparticles (SLN) to overcome the poor efficiency problem, but the expression of the target gene depended on the cells and administration routes [69]; (5) The effect of gene therapy is also dependent on the stage of disease. Researchers demonstrated that greater improvements of the retina’s morphology and function are generally seen in younger mice than adult mice [70]. These results emphasized the importance of early detection in RP patients.

## 3. The Progress of Stem Cell Treatment

Several studies showed that BMSCs could differentiate into chondrocytes, fibroblasts, adipocytes, cardiomyocytes, and skeletal muscle cells *in vitro* [71,72,73], (also see Table 2 the stem cell therapy used in other tissues).

The differentiation of BMSCs into neuronal cells had great concern for a long time. The experiments confirmed that BMSCs could differentiate into neuron-like cells in the appropriate induction medium or cytokines *in vitro* [74,75]. *In vivo* experiments showed that BMSCs could differentiate into neuron-like cells and the way of neurogenesis, maturation, and migration was also the same as in neural stem cells. In the field of ophthalmology, BMSCs could be induced to differentiate into corneal epithelial cells, retinal photoreceptor-like cells, *etc.* [44,76].

**Table 2:** Stem cell therapy used in other tissues.

Function	Types of Stem Cells	Application
**Antenatal Diagnosis [77]**	Menstrual-derived stem cells	While more insight on their immunomodulatory and diagnostic properties is needed, the impact of clinical and epidemiological factors, such as age, use of contraceptives, or hormonal status still requires further investigations to properly assess their current and future use in clinical application and diagnosis.
**Protection and Repair the Developing Brain [78]**	Cord blood and amnion epithelial derived cells	Perinatal brain injury may result from acute or chronic insults sustained during fetal development, during the process of birth, or in the newborn period. The stem cells have the potential for transplantation to the newborn where brain injury is diagnosed or even suspected.
**Malignant Glioma [79]**	Neural stem cells, and multipotent mesenchymal stromal cells	Tumour cell-derived substances and factors associated with tumour-induced inflammation and tumour neovascularisation can specifically attract stem cells to invasive gliomas. Injected stem cells engineered to produce anti-tumour substances showed strong therapeutic effects.
**Liver and Pancreas [80]**	Hepatic stem/progenitor cells, mesenchymal stemcells and hematopoietic stem cells	Hepatic stem/progenitors cells were transplanted into the hepatic artery of patients with various liver diseases and immunosuppression was not required MSCs have demonstrated significant effects through paracrine signaling of trophic and immunomodulatory factors.
**Musculoskeletal Regeneration [81]**	Embryonic stem cells induced-pluripotent stem cells adult tissue-derived mesenchymal stem cells	It holds promise in treating numerous musculoskeletal diseases and injuries. The combination of biomaterial scaffolds and bioreactors provides methods to create an environment for stem cells.
**Amyotrophic Lateral Sclerosis [82]**	Mesenchymal/blood-derived stem cells	Amyotrophic lateral sclerosis (ALS) is a devastating neurodegenerative disorder that is characterized by progressive degeneration of motor neurons in the cortex, brainstem and spinal cord. There is more rationale for using stem cells as support cells for dying motor neurons as they are already connected to the muscle.
**Bladder Dysfunction [83]**	Adipose derived stem cells, bone marrow stem cells, and skeletal muscle derived stem cells	The therapeutic efficacy of stem cells was originally thought to be derived from their ability to differentiate into various cell types. The main mechanisms of stem cells to reconstitute or restore bladder dysfunction are migration, differentiation, and paracrine effects.
**Neuropathic Pain [84]**	Human mesenchymal stem/stromal cells	Human mesenchymal stem/stromal cells produce a large variety, and the secretion of neurotrophic factors by stem cells provides neuroprotection and neuroregenerative effects of trophic factors.
**Kidney [85]**	Renal stem/progenitor cells	Renal stem/progenitor system is present in the tubules, interstitium, and glomeruli of the adult kidney and functions as the main drivers of kidney regenerative responses after injury by secreting renotropic factors.
**Cardiac Disease [86]**	Cardiac progenitor cells, embryonic stem cells, induced pluripotent stem cells, bone marrow stem cells and mesenchymal stem cells	Mainly applied in acute myocardial infarction and ischemic cardiomyopathy, * in vivo* transplanted stem cells can proliferate and differentiate into cardiomyocytes, endothelial cells, or smooth muscle cells, clinical trials showed a reassuring safety profile and suggest functional benefits.
**Type 1 Diabetes [87]**	Embryonic stem cells, induced pluripotent stem cells, bone marrow-derived hematopoietic stem cells, and multipotent mesenchymal stromal cells derived from bone marrow, umbilical cord blood, and adipose tissue	Stem cell-based strategies to restore glycometabolic and immune homeostasis are based on the intrinsic regenerative capacity as well as the immunomodulatory potential of stem cells. The regenerative capacity can be harnessed to make available a self-replenishing supply of glucose-responsive insulin-producing cells for transplantation.
**Plastic Surgery [88]**	Mesenchymal stem cells, embryonic stem cells, induced pluripotent stem cell	Stem cells mainly used in soft tissue augmentation and regeneration, reconstructing bony defects, cartilage formation, wound healing, skin rejuvenation and peripheral nerve regeneration.

In 2002, Otani *et al.* published the article about the use of BMSCs in the field of ophthalmology: They used mice bone marrow-derived endothelial precursor cells (EPCs) to inject into the vitreous cavity of mice and proved that the cell can participate in the formation of blood vessels in the retina [22].

After that, researchers tried different types of cells and methods, and some of them could promote photoreceptor cell survival [45], the stem cells even could differentiate into retinal cells and improve retinal function [44,89]. But most experiments were carried on animals. They provided evidence of safety and feasibility of stem cells transplantation, so several clinical trials were conducted. Encouraging progess prompted phase I and phase II clinical trials in which autologous bone marrow-derived mononuclear cells were injected into the vitreous of RP patients. Laongsri Atchaneeyasakul *et al.*, and Sachin Jamadar *et al.*, also prompted a phase I and phase I/II in RP patients, the effect is still be observed.

In 2010 and 2011, the ACT company in United States received two FDA approvals for using hESC to treat macular degeneration diseases in phase I/II clinical trials. They transplanted hESC-derived RPE into the subretinal space of patients. After surgery, structural evidence confirmed cells had attached and continued to persist during the study and they did not identify any signs of hyper proliferation, abnormal growth, or immune mediated transplant rejection during the first 4 months; during the observation period neither patient lost vision [90].

Jost and his colleagues carried out a clinical trial about injection of autologous bone-marrow-derived cells into the vitreous cavity on 3 persons, who suffered at the end stage of the ocular diseases. At the end of follow-up (no more than 1 year), visual acuity was unchanged for all patients. But this report suggests that the injection of stem cells into the vitreous cavity is technically feasible. Possible reasons for unchanged visual acuity might be that at the end stage of the ocular diseases the cells can not give full play to eye [47,48]. Studies evaluated the pharmacokinetics of ciliary neurotrophic factor (CNTF) delivered over a period of up to 2 years by an intraocular encapsulated cell technology (ECT) implant in patients with RP and geographic atrophy (GA). Implants produced CNTF consistently over a 2 year period. In both GA and RP clinical studies, no serious adverse events occured over the course of either the 12- or 24-month study and the clinical trial results provided evidence that sustained intraocular delivery of CNTF protected the retina from degeneration [91]. Rubens and his colleagues carried out a phase I clinical trial including 3 patients with retinitis pigmentosa and 2 patients with cone–rod dystrophy; they were injected with autologous bone marrow-derived mononuclear cells into the vitreous cavity; after 10 months, no detectable structural or functional toxicity was observed [92]. They also demonstrated that autologous bone marrow-derived mononuclear cells had a positive effect on cystoid macular oedema (CMO) associated with RP. The improvement of the oedema led to an improvement in visual acuity and an improvement in macular sensitivity, as measured by the microperimetry test [93]. Thus, the most suitable stage for RP patients to receive the cells should be taken into consideration. Registered clinical trials on mesenchymal stem cells for retinal diseases are shown in Table 3.

From the results of animal experiments to clinical trials, we predict that the use of stem cells for retinitis pigmentosa is a promising treatment.

**Table 3:** Registered clinical trials on mesenchymal stem cells for retinal diseases. Information obtained from ClinicalTrials.gov.

Identifier	Country	Study	Phase of Trial	Intervention	Disease	Cells
NCT01914913	India	Clinical study to evaluate safety and efficacy of stem cell Therapy in Retinitis Pigmentosa	Phase 1, Phase 2	Transfer of mesenchymal stem cell	RP	Bone marrow and umbilical cord derived mesenchymal stem cell
NCT01068561 NCT01560715	Brazil	Autologous bone marrow-derived stem cells transplantation for retinitis pigmentosa	Phase 1, Phase 2	Intravitreal injection	RP	Autologous bone marrow stem cells
NCT01531348	Thailand	Feasibility and safety of adult human bone marrow-derived mesenchymal stem cells by intravitreal injection in patients with retinitis pigmentosa	Phase 1	Intravitreal injection	RP	Adult human bone marrow-derived mesenchymal stem cells
NCT01736059	USA	Clinical trial of autologous intravitreal bone-marrow CD34+ stem cells for retinopathy	Phase 1	Intravitreal injection	Dry AMD; DR; RVO; RP; Hereditary macular degeneration	CD34+ bone marrow stem cells
NCT01920867	USA	Stem cell ophthalmology treatment study		Retrobulbar Subtenon Intravenous Intravitreal Intraocular	Retinal disease; macular degeneration; hereditary retinal Dystrophy optic nerve disease; glaucoma	Autologous bone marrow derived stem cells

AMD, Age-related Macular Degeneration; DR, diabetic retinopathy; RVO, retinal vein occlusion.

## 4. Mechanisms of Stem Cell Therapy

The mechanisms of cell transplantation stem cells therapy for retinal degenerative diseases: (1) cell replacement uses healthy stem cells to replace degenerated cells; (2) nutritional support transplants healthy stem cells to promote the survival of surrounding cells by secreting the trophic factors; (3) protection of the retinal blood vessels and cones by upregulating antiapoptotic genes; (4) promotion of new synaptic connections.

### 4.1. Cell Replacement

Researchers hold the opinion that transplanted stem cells can differentiate into retinal cells and integrate into the retina of patients, and that the differentiated stem cells replace the apoptotic or injured retinal cells [94].

Many studies demonstrated that the stem cells have the potential to differentiate into photoreceptor cells mainly by three methods, including gene transduction, taking advantage of cell carrier and using cytokines. One finding suggested noggin-transduced hBMSCs produced more photoreceptor cells. The *noggin* gene was transferred into adult hBMSCs, these cells expressed multiple markers including rhodopsin after differentiation [95]. Nadri and colleagues used nanofibrous scaffolds as cell carriers to shape the photoreceptor-like cells from conjunctiva mesenchymal stem cells [96]. They also used amniotic membranes to induce mesenchymal stem cells from a trabecular meshwork to photoreceptor-like cells [92]. Researchers found that after incubation of rat BMSCs with epidermal growth factor (EGF), taurine or activin A, 20%–32% of the cells expressed rhodopsin [44].

Many studies showed that the stem cells can differentiate into photoreceptor cells and/or RPE in the microenvironment of retinal degeneration.

Kicic *et al.* induced BMSCs that expressed rhodopsin. The RCS rats were transplanted with those cells into the subretinal space, after 2 weeks the cells were mainly located in the outer nuclear layer of the retina and also expressed rhodopsin. The results also have shown that differentiated cells can attract synaptic vesicles, which indicates that it is possible to improve the visual signal pathway [44]. Tomita *et al.*, found that after transplantation of BMSCs into the vitreous cavity of rats with damaged retinas, the BMSCs could protect the normal structure of the outer nuclear layer (ONL) of the retina, and also express surface antigens of astrocytes (glial fibrillary acidic protein, GFAP) and retinal photoreceptor cell (rhodopsin) [97]. Researchers also demonstrated that cells can express rhodopsin in rat BMSCs transplanted into the subretinal space of sodium iodate-injected rats [98]. Arnhold *et al.*, also proved that BMSCs transplanted into the subretinal space of the rhodopsin knockout mouse, can express rhodopsin [99]. Huo proved that MSCs can survive mainly in the outer layer of retina in the microenvironment of retinal degeneration and differentiate forward the RPE cells and photoreceptors [100].

Mouse embryonic stem (ES) cells also can differentiate into RPE-like cells *in vitro* and then restore retinal function in a mouse model for retinitis pigmentosa by transplanting the cells into the subretinal space. Based on this finding, it was proved that ES cells can differentiate, morphologically and functionally, into RPE-like cells [30]. Colozza and his colleagues proved that after transplantation of progenitor stem cells, the cells can be stimulated to become replacement photoreceptors and supportive outer retina cells can theoretically lead to treatments that restore visual function [101].

Many studies showed that stem cells have the potential for differentiation into retinal cells *in vitro* or *in vivo* and even participate in signal pathways, and further improve retinal function.

### 4.2. Nutritional Support

Researchers have assumed that the function of transplanted cells is releasing diffusible factors and acting as a local cell delivery system for trophic factors.

A recent study investigated whether BMSCs secrete factors to promote photoreceptor cell survival. *In vivo*, subretinal transplantation of BMSCs of Pcrx2K-lacZ transgenic mice could promote photoreceptor survival in RCS rats. *In vitro*, they demonstrated that the conditioned medium of the MSCs delayed photoreceptor cell apoptosis. These results suggest that BMSCs are a useful cell source for secreting factors and that these factors can promote photoreceptor cell survival [45]. When RCS were received BMSCs by tail vein at an age before major photoreceptor loss, visual function was significantly preserved; semi-quantitative RT-PCR analysis indicated there was up-regulation of growth factors and immunohistochemistry revealed that there was an increase in neurotrophic factors. This study thus demonstrated that stem cells can secrete neurotrophic factors to promote photoreceptor cell survival [102].

Studies using a co-culture system between rat BMSCs and hippocampal neurons without any direct contact have shown that BMSCs are capable of supporting survival of hippocampal neurons. This directly proved that the release of nutritional substances from BMSCs was necessary for neuronal survival [103].

### 4.3. Protection of the Retinal Blood Vessels and Cones

RP is characterized by degenerative changes in the photoreceptors, primarily rods and secondarily cones. Loss of retinal vasculature is a presumed metabolic consequence of photoreceptor degeneration [104].

Using a fraction of bone marrow derived stem cells which contained endothelial precursors can rescue retinal blood vessels that would ordinarily completely degenerate, a dramatic neurotrophic rescue effect is also observed. Microarray analysis of rescued retinas demonstrates significant up-regulation of many antiapoptotic genes [22,105]. The BMSCs were injected to RCS by tail vein at an age before major photoreceptor loss, and after an extended period, the number of pathological vascular complexes (abnormal vessels associated with migrating pigment epithelium cells) and area of vascular leakage that would ordinarily develop were dramatically reduced [102].

Researchers observed that the bone marrow-derived stem cells (whether derived from defective mice or wild-type mice), after transplantation can preserve cone vision. It is suggested that in humans, the patient’s own, or a normal person’s bone marrow cells may provide potential cone neuroprotection to preserve central vision [104].

### 4.4. Promotion Synaptic Connections

MacLaren and Bartsch have showed that in a specific period (donor cells were taken from the developing retina at a time coincident with the peak of rod genesis) stem cells from mice can integrate into the adult or degenerating mice retina, form synaptic connections with retinopathy and effectively improve visual function [26,106].

## 5. Problems to Be Solved

Despite the recent progress made by stem cell transplantation therapy for retinal degeneration, many challenges remain.

### 5.1. Immunity Effects of Subretinal Space in Stem Cell Therapy

The retina is physically isolated from the systemic immune system and has low immunogenicity. But immune rejection is still treated as a major barrier, especially in humans, whose genetic makeup is more heterogeneous than experimental models [107,108]. After stem cells transplantation into the subretinal space, systemic immunity appeared to exert a slow influence. Transplantation of RPE cells was performed in two strains of RCS rats, which have incompatible major histocompatibility complex (MHC) haplotypes. Despite the absence of acute immune rejection, the increased loss of photoreceptor cells showed chronic rejection [109].

But some researchers hold another opinion, allogeneic transplants from animals prove that the subretinal region is immune privileged because of the presence of the blood–brain and blood–retinal barriers, making it possible to transplant cell into the human retina. Human embryonic stem cell (shESCs) were transplanted into the subretinal and epiretinal space of mice, then the mice were examined for survival of hESCs. It was shown that the cells survived in a xenogenic environment without immunosuppression as long as the blood–retinal barrier was not breached by the transplantation procedure [110].

### 5.2. Low Rate of Cell Survival and Migration

A direct correlation between grafted cell density and numbers of surviving photoreceptor cells is strong evidence for the rescue effect. But a low rate of transplanted cell survival is a major problem in stem cell therapy [49].

Although some cells will integrate into the host retina, a high percentage of dissociated transplanted photoreceptor precursors remain in the subretinal space without contact to the host retina [106,110,111].

The natural barrier of stem cell migration is the extracellular matrix (ECM) [112]. Researchers have adopted a variety of ways to improve transplantation efficiency, such as using laser injury to disrupt the natural physical barriers at the outer retina, or disrupting the outer limiting membrane junctional proteins (Crb1 and ZO-1) to increase integration [113,114,115].

Researchers suggest that the OLM presents a physical barrier to photoreceptor integration following transplantation into the subretinal space in the adult mouse. Pharmacological disruption of the outer limiting membrane leads to increased retinal integration of transplanted photoreceptor precursors [116]

### 5.3. Biosafety Issues

Firstly, it is important to solve stem cells massive amplification problem.

Secondly, differentiated stem cell therapy must meet the criteria in clinical applications, such as: Cells can be provided in unlimited quantities, produced cells have no pathogens, no differences among batches, ensure every stem cell can differentiate into the desired cells for eye treatment.

Thirdly, transplants should not form tumors or be harmful to recipients; mechanisms of action need to be clarified.

Fourthly, it must be determined which kind of stem cell is most suitable for stem cell therapy, the dosage, and the most suitable stage for RP patients to received the cells.

## 6. Outlook for the Treatment of Retinitis Pigmentosa

The studies using stem cells in animal models and clinical trials have demonstrated promising results and provide encouraging groundwork on which to continue research in the pursuit of optimal cellular therapies for the treatment of RP. Clinical trials suggest that stem cell injection is technically feasible and has no detectable structural or functional toxicity in the long term. With the progress of study, more and more treatment methods are constantly improved. The current treatment methods and means are mainly through the inhibition of apoptosis, and protection, replenishment or repair of the RPE and photoreceptor cells. Improvement of their comprehensive treatment programs and treatment effectiveness is the direction of our future efforts.

## References

[1] Girman S.V., Wang S., Lund R.D. (2003). Cortical visual functions can be preserved by subretinal RPE cell grafting in RCS rats. Vis. Res..

[2] Rakoczy E.P., Kiel C., McKeone R., Stricher F., Serrano L. (2011). Analysis of disease-linked rhodopsin mutations based on structure, function, and protein stability calculations. J. Mol. Biol..

[3] Bramall A.N., Wright A.F., Jacobson S.G., McInnes R.R. (2010). The genomic, biochemical, and cellular responses of the retina in inherited photoreceptor degenerations and prospects for the treatment of these disorders. Annu. Rev. Neurosci..

[4] Lambiase A., Aloe L. (1996). Nerve growth factor delays retinal degeneration in C3H mice. Graefes Arch. Clin. Exp. Ophthalmol..

[5] Radu R.A., Yuan Q., Hu J., Peng J.H., Lloyd M., Nusinowitz S., Bok D., Travis G.H. (2008). Accelerated accumulation of lipofuscin pigments in the RPE of a mouse model for ABCA4-mediated retinal dystrophies following vitamin A supplementation. Investig. Ophthalmol. Vis. Sci..

[6] Tsubura A., Yuri T., Yoshizawa K., Uehara N., Takada H. (2009). Role of fatty acids in malignancy and visual impairment: Epidemiological evidence and experimental studies. Histol. Histopathol..

[7] Nguyen C.T.O., Vingrys A.J., Bui B.V. (2008). Dietary ω-3 fatty acids and ganglion cell function. Investig. Ophthalmol. Vis. Sci..

[8] Vingolo E.M., Rocco M., Grenga P., Salvatore S., Pelaia P. (2008). Slowing the degenerative process, long lasting effect of hyperbaric oxygen therapy in retinitis pigmentosa. Graefes Arch. Clin. Exp. Ophthalmol..

[9] Lenzi L., Coassin M., Lambiase A., Bonini S., Amendola T., Aloe L. (2005). Effect of exogenous administration of nerve growth factor in the retina of rats with inherited retinitis pigmentosa. Vis. Res..

[10] Schallenberg M., Charalambous P., Thanos S. (2012). GM-CSF protects rat photoreceptors from death by activating the SRC-dependent signalling and elevating anti-apoptotic factors and neurotrophins. Graefes Arch. Clin. Exp..

[11] Cideciyan A.V., Jacobson S.G., Beltran W.A., Sumaroka A., Swider M., Iwabe S., Roman A.J., Olivares M.B., Schwartz S.B., Komaromy A.M. (2013). Human retinal gene therapy for Leber congenital amaurosis shows advancing retinal degeneration despite enduring visual improvement. Proc. Natl. Acad. Sci. USA.

[12] Wert K.J., Davis R.J., Sancho-Pelluz J., Nishina P.M., Tsang S.H. (2013). Gene therapy provides long-term visual function in a pre-clinical model of retinitis pigmentosa. Hum. Mol. Genet..

[13] Petrs-Silva H., Linden R. (2014). Advances in gene therapy technologies to treat retinitis pigmentosa. Clin. Ophthalmol..

[14] Berger W., Kloeckener-Gruissem B., Neidhardt J. (2010). The molecular basis of human retinal and vitreoretinal diseases. Prog. Retin. Eye Res..

[15] Anasagasti A., Irigoyen C., Barandika O., Lopez de Munain A., Ruiz-Ederra J. (2012). Current mutation discovery approaches in Retinitis Pigmentosa. Vis. Res..

[16] Boughman J.A., Fishman G.A. (1983). A genetic analysis of retinitis pigmentosa. Br. J. Ophthalmol..

[17] Conlon T.J., Deng W.T., Erger K., Cossette T., Pang J.J., Ryals R., Clement N., Cleaver B., McDoom I., Boye S.E. (2013). Preclinical potency and safety studies of an AAV2-mediated gene therapy vector for the treatment of MERTK associated retinitis pigmentosa. Hum. Gene Ther. Clin. Dev..

[18] Lawrence J.M., Sauve Y., Keegan D.J., Coffey P.J., Hetherington L., Girman S., Whiteley S.J., Kwan A.S., Pheby T., Lund R.D. (2000). Schwann cell grafting into the retina of the dystrophic RCS rat limits functional deterioration. Royal College of Surgeons. Investig. Ophthalmol. Vis. Sci..

[19] McGill T.J., Cottam B., Lu B., Wang S., Girman S., Tian C., Huhn S.L., Lund R.D., Capela A. (2012). Transplantation of human central nervous system stem cells—Neuroprotection in retinal degeneration. Eur. J. Neurosci..

[20] Huo S.J., Li Y.C., Xie J., Li Y., Raisman G., Zeng Y.X., He J.R., Weng C.H., Yin Z.Q. (2012). Transplanted olfactory ensheathing cells reduce retinal degeneration in royal college of surgeons rats. Curr. Eye Res..

[21] Tian C., Zhao T., Zeng Y., Yin Z.Q. (2011). Increased muller cell de-differentiation after grafting of retinal stem cell in the sub-retinal space of royal college of surgeons rats. Tissue Eng. Part A.

[22] Otani A., Kinder K., Ewalt K., Otero F.J., Schimmel P., Friedlander M. (2002). Bone marrow-derived stem cells target retinal astrocytes and can promote or inhibit retinal angiogenesis. Nat. Med..

[23] Haruta M., Sasai Y., Kawasaki H., Amemiya K., Ooto S., Kitada M., Suemori H., Nakatsuji N., Ide C., Honda Y. (2004). *In vitro* and *in vivo* characterization of pigment epithelial cells differentiated from primate embryonic stem cells. Investig. Ophthalmol. Vis. Sci..

[24] Aramant R.B., Seiler M.J., Ball S.L. (1999). Successful cotransplantation of intact sheets of fetal retina with retinal pigment epithelium. Investig. Ophthalmol. Vis. Sci..

[25] Gouras P., Flood M.T., Kjedbye H., Bilek M.K., Eggers H. (1985). Transplantation of cultured human retinal epithelium to Bruch’s membrane of the owl monkey’s eye. Curr. Eye Res..

[26] MacLaren R.E., Pearson R.A., MacNeil A., Douglas R.H., Salt T.E., Akimoto M., Swaroop A., Sowden J.C., Ali R.R. (2006). Retinal repair by transplantation of photoreceptor precursors. Nature.

[27] Qiu G., Seiler M.J., Mui C., Arai S., Aramant R.B., de Juan E., Sadda S. (2005). Photoreceptor differentiation and integration of retinal progenitor cells transplanted into transgenic rats. Exp. Eye Res..

[28] West E.L., Pearson R.A., Barker S.E., Luhmann U.F.O., Maclaren R.E., Barber A.C., Duran Y., Smith A.J., Sowden J.C., Ali R.R. (2010). Long-term survival of photoreceptors transplanted into the adult murine neural retina requires immune modulation. Stem Cells.

[29] Yanai A., Laver C., Joe A.W., Gregory-Evans K. Efficient production of photoreceptor precursor cells from human embryonic stem cells. Methods Mol. Biol..

[30] Wang N.K., Tosi J., Kasanuki J.M., Chou C.L., Kong J., Parmalee N., Wert K.J., Allikmets R., Lai C.C., Chien C.L. (2010). Transplantation of reprogrammed embryonic stem cells improves visual function in a mouse model for retinitis pigmentosa. Transplantation.

[31] Idelson M., Alper R., Obolensky A., Ben-Shushan E., Hemo I., Yachimovich-Cohen N., Khaner H., Smith Y., Wiser O., Gropp M. (2009). Directed differentiation of human embryonic stem cells into functional retinal pigment epithelium cells. Cell Stem Cell.

[32] Reubinoff B.E., Pera M.F., Fong C.Y., Trounson A., Bongso A. (2000). Embryonic stem cell lines from human blastocysts: Somatic differentiation *in vitro*. Nat. Biotechnol..

[33] Cowan C.A., Klimanskaya I., McMahon J., Atienza J., Witmyer J., Zucker J.P., Wang S.P., Morton C.C., McMahon A.P., Powers D. (2004). Derivation of embryonic stem-cell lines from human blastocysts. N. Engl. J. Med..

[34] Yu J.Y., Vodyanik M.A., Smuga-Otto K., Antosiewicz-Bourget J., Frane J.L., Tian S., Nie J., Jonsdottir G.A., Ruotti V., Stewart R. (2007). Induced pluripotent stem cell lines derived from human somatic cells. Science.

[35] Jin Z.B., Okamoto S., Xiang P., Takahashi M. (2012). Integration-free induced pluripotent stem cells derived from retinitis pigmentosa patient for disease modeling. Stem Cell Trans. Med..

[36] Tucker B.A., Mullins R.F., Streb L.M., Anfinson K., Eyestone M.E., Kaalberg E., Riker M.J., Drack A.V., Braun T.A., Stone E.M. (2013). Patient-specific iPSC-derived photoreceptor precursor cells as a means to investigate retinitis pigmentosa. Elife.

[37] Li Y., Tsai Y.T., Hsu C.W., Erol D., Yang J., Wu W.H., Davis R.J., Egli D., Tsang S.H. (2012). Long-term safety and efficacy of human-induced pluripotent stem cell (iPS) grafts in a preclinical model of retinitis pigmentosa. Mol. Med..

[38] Puzio-Kuter A.M., Levine A.J. (2009). Stem cell biology meets p53. Nat. Biotechnol..

[39] Friedenstein A.J., Gorskaja J.F., Kulagina N.N. (1976). Fibroblast precursors in normal and irradiated mouse hematopoietic organs. Exp. Hematol..

[40] In’t Anker P.S., Scherjon S.A., Kleijburg-van der Keur C., de Groot-Swings G.M., Claas F.H., Fibbe W.E., Kanhai H.H. (2004). Isolation of mesenchymal stem cells of fetal or maternal origin from human placenta. Stem Cells.

[41] Lee O.K., Kuo T.K., Chen W.M., Lee K.D., Hsieh S.L., Chen T.H. (2004). Isolation of multipotent mesenchymal stem cells from umbilical cord blood. Blood.

[42] Ghorbani A., Jalali S.A., Varedi M. (2014). Isolation of adipose tissue mesenchymal stem cells without tissue destruction: A non-enzymatic method. Tissue Cell.

[43] Secunda R., Vennila R., Mohanashankar A.M., Rajasundari M., Jeswanth S., Surendran R. Isolation, expansion and characterisation of mesenchymal stem cells from human bone marrow, adipose tissue, umbilical cord blood and matrix: A comparative study. Cytotechnology.

[44] Kicic A., Shen W.Y., Wilson A.S., Constable I.J., Robertson T., Rakoczy P.E. (2003). Differentiation of marrow stromal cells into photoreceptors in the rat eye. J. Neurosci..

[45] Inoue Y., Iriyama A., Ueno S., Takahashi H., Kondo M., Tamaki Y., Araie M., Yanagi Y. (2007). Subretinal transplantation of bone marrow mesenchymal stem cells delays retinal degeneration in the RCS rat model of retinal degeneration. Exp. Eye Res..

[46] Siqueira R.C., Messias A., Voltarelli J.C., Scott I.U., Jorge R. (2011). Intravitreal injection of autologous bone marrow-derived mononuclear cells for hereditary retinal dystrophy a phase I trial. Retina.

[47] Jonas J.B., Witzens-Harig M., Arseniev L., Ho A.D. (2008). Intravitreal autologous bone marrow-derived mononuclear cell transplantation: A feasibility report. Acta Ophthalmol..

[48] Jonas J.B., Witzens-Harig M., Arseniev L., Ho A.D. (2010). Intravitreal autologous bone-marrow-derived mononuclear cell transplantation. Acta Ophthalmol..

[49] Klassen H.J., Ng T.F., Kurimoto Y., Kirov I., Shatos M., Coffey P., Young M.J. (2004). Multipotent retinal progenitors express developmental markers, differentiate into retinal neurons, and preserve light-mediated behavior. Investig. Ophthalmol. Vis. Sci..

[50] Ruitenberg M.J., Vukovic J., Sarich J., Busfield S.J., Plant G.W. (2006). Olfactory ensheathing cells: Characteristics, genetic engineering, and therapeutic potential. J. Neurotrauma.

[51] Ramon-Cueto A., Avila J. (1998). Olfactory ensheathing glia: Properties and function. Brain Res. Bull..

[52] Huo S.J., Li Y., Raisman G., Yin Z.Q. (2011). Transplanted olfactory ensheathing cells reduce the gliotic injury response of Muller cells in a rat model of retinitis pigmentosa. Brain Res..

[53] Gamm D.M., Wang S., Lu B., Girman S., Holmes T., Bischoff N., Shearer R.L., Sauve Y., Capowski E., Svendsen C.N. (2007). Protection of visual functions by human neural progenitors in a rat model of retinal disease. PLoS One.

[54] Wang S., Girman S., Lu B., Bischoff N., Holmes T., Shearer R., Wright L.S., Svendsen C.N., Gamm D.M., Lund R.D. (2008). Long-term vision rescue by human neural progenitors in a rat model of photoreceptor degeneration. Investig. Ophthalmol. Vis. Sci..

[55] Behrstock S., Ebert A., McHugh J., Vosberg S., Moore J., Schneider B., Capowski E., Hei D., Kordower J., Aebischer P. (2006). Human neural progenitors deliver glial cell line-derived neurotrophic factor to parkinsonian rodents and aged primates. Gene Ther..

[56] Klein S.M., Behrstock S., McHugh J., Hoffmann K., Wallace K., Suzuki M., Aebischer P., Svendsen C.N. (2005). GDNF delivery using human neural progenitor cells in a rat model of ALS. Hum. Gene Ther..

[57] Dinculescu A., Min S.H., Deng W.T., Li Q., Hauswirth W.W. (2014). Gene therapy in the RD6 mouse model of retinal degeneration. Adv. Exp. Med. Biol..

[58] Millington-Ward S., Chadderton N., O’Reilly M., Palfi A., Goldmann T., Kilty C., Humphries M., Wolfrum U., Bennett J., Humphries P. (2011). Suppression and replacement gene therapy for autosomal dominant disease in a murine model of dominant retinitis pigmentosa. Mol. Ther..

[59] Mao H.Y., Gorbatyuk M.S., Rossmiller B., Hauswirth W.W., Lewin A.S. (2012). Long-term rescue of retinal structure and function by rhodopsin rna replacement with a single adeno-associated viral vector in *P23H RHO* transgenic mice. Hum. Gene Ther..

[60] Liu M.M., Tuo J.S., Chan C.C. (2011). Gene therapy for ocular diseases. Br. J. Ophthalmol..

[61] Tosi J., Sancho-Pelluz J., Davis R.J., Hsu C.W., Wolpert K.V., Sengillo J.D., Lin C.S., Tsang S.H. (2011). Lentivirus-mediated expression of cDNA and shRNA slows degeneration in retinitis pigmentosa. Exp. Biol. Med..

[62] Pang J.J., Boye S.L., Kumar A., Dinculescu A., Deng W., Li J., Li Q., Rani A., Foster T.C., Chang B. (2008). AAV-mediated gene therapy for retinal degeneration in the RD10 mouse containing a recessive PDEβ mutation. Investig. Ophthalmol. Vis. Sci..

[63] Pang J.J., Dai X.F., Boye S.E., Barone I., Boye S.L., Mao S., Everhart D., Dinculescu A., Liu L., Umino Y. (2011). Long-term retinal function and structure rescue using capsid mutant AAV8 vector in the RD10 mouse, a model of recessive retinitis pigmentosa. Mol. Ther..

[64] Hartong D.T., Berson E.L., Dryja T.P. (2006). Retinitis pigmentosa. Lancet.

[65] Loewen C.J.R., Moritz O.L., Molday R.S. (2001). Molecular characterization of peripherin-2 and Rom-1 mutants responsible for digenic retinitis pigmentosa. J. Biol. Chem..

[66] Duno M., Wibrand F., Baggesen K., Rosenberg T., Kjaer N., Frederiksen A.L. (2013). A novel mitochondrial mutation m.8989G>C associated with neuropathy, ataxia, retinitis pigmentosa—The NARP syndrome. Gene.

[67] Schlichtenbrede F.C., MacNeil A., Bainbridge J.W., Tschernutter M., Thrasher A.J., Smith A.J., Ali R.R. (2003). Intraocular gene delivery of ciliary neurotrophic factor results in significant loss of retinal function in normal mice and in the Prph2Rd2/Rd2 model of retinal degeneration. Gene Ther..

[68] Manic G., Maurin-Marlin A., Galluzzi L., Subra F., Mouscadet J.F., Bury-Mone S. (2012). 3' Self-inactivating long terminal repeat inserts for the modulation of transgene expression from lentiviral vectors. Hum. Gene Ther. Method.

[69] Delgado D., del Pozo-Rodriguez A., Solinis M.A., Aviles-Triqueros M., Weber B.H.F., Fernandez E., Gascon A.R. (2012). Dextran and protamine-based solid lipid nanoparticles as potential vectors for the treatment of X-Linked juvenile retinoschisis. Hum. Gene Ther..

[70] Sarra G.M., Stephens C., de Alwis M., Bainbridge J.W.B., Smith A.J., Thrasher A.J., Ali R.R. (2001). Gene replacement therapy in the retinal degeneration slow (RDs) mouse: The effect on retinal degeneration following partial transduction of the retina. Hum. Mol. Genet..

[71] Gimble J.M., Zvonic S., Floyd Z.E., Kassem M., Nuttall M.E. (2006). Playing with bone and fat. J. Cell. Biochem..

[72] Dezawa M., Kanno H., Hoshino M., Cho H., Matsumoto N., Itokazu Y., Tajima N., Yamada H., Sawada H., Ishikawa H. (2004). Specific induction of neuronal cells from bone marrow stromal cells and application for autologous transplantation. J. Clin. Investig..

[73] Gregory C.A., Prockop D.J., Spees J.L. (2005). Non-hematopoietic bone marrow stem cells: Molecular control of expansion and differentiation. Exp. Cell Res..

[74] Woodbury D., Schwarz E.J., Prockop D.J., Black I.B. (2000). Adult rat and human bone marrow stromal cells differentiate into neurons. J. Neurosci. Res..

[75] Yang J.D., Cheng H., Wang J.C., Feng X.M., Li Y.N., Xiao H.X. The isolation and cultivation of bone marrow stem cells and evaluation of differences for neural-like cells differentiation under the induction with neurotrophic factors. Cytotechnology.

[76] Gu S., Xing C., Han J., Tso M.O., Hong J. (2009). Differentiation of rabbit bone marrow mesenchymal stem cells into corneal epithelial cells *in vivo* and *ex vivo*. Mol. Vis..

[77] Khoury M., Alcayaga-Miranda F., Illanes S.E., Figueroa F.E. (2014). The promising potential of menstrual stem cells for antenatal diagnosis and cell therapy. Front. Immunol..

[78] Castillo-Melendez M., Yawno T., Jenkin G., Miller S.L. (2013). Stem cell therapy to protect and repair the developing brain: A review of mechanisms of action of cord blood and amnion epithelial derived cells. Front. Neurosci..

[79] Bexell D., Svensson A., Bengzon J. (2013). Stem cell-based therapy for malignant glioma. Cancer Treat. Rev..

[80] Lanzoni G., Oikawa T., Wang Y.F., Cui C.B., Carpino G., Cardinale V., Gerber D., Gabriel M., Dominguez-Bendala J., Furth M.E. (2013). Concise review: Clinical programs of stem cell therapies for liver and pancreas. Stem Cells.

[81] Brown P.T., Handorf A.M., Jeon W.B., Li W.J. (2013). Stem cell-based tissue engineering approaches for musculoskeletal regeneration. Curr. Pharm. Des..

[82] Thomsen G.M., Gowing G., Svendsen S., Svendsen C.N. The past, present and future of stem cell clinical trials for ALS. Exp. Neurol..

[83] Kim J.H., Lee S.R., Song Y.S., Lee H.J. (2013). Stem cell therapy in bladder dysfunction: Where are we? and where do we have to go?. Biomed. Res. Int..

[84] Fortino V.R., Pelaez D., Cheung H.S. (2013). Concise review: Stem cell therapies for neuropathic pain. Stem Cell Trans. Med..

[85] Maeshima A., Nakasatomi M., Nojima Y. (2014). Regenerative medicine for the kidney: Renotropic factors, renal stem/progenitor cells, and stem cell therapy. Biomed. Res. Int..

[86] Kwon S.U., Yeung A.C., Ikeno F. (2013). The role of large animal studies in cardiac regenerative therapy concise review of translational stem cell research. Korean Circ. J..

[87] Chhabra P., Brayman K.L. (2013). Stem cell therapy to cure type 1 diabetes: From hype to hope. Stem Cells Trans. Med..

[88] Salibian A.A., Widgerow A.D., Abrouk M., Evans G.R. (2013). Stem cells in plastic surgery: A review of current clinical and translational applications. Arch. Plast. Surg..

[89] Lu B., Wang S., Girman S., McGill T., Ragaglia V., Lund R. (2010). Human adult bone marrow-derived somatic cells rescue vision in a rodent model of retinal degeneration. Exp. Eye Res..

[90] Schwartz S.D., Hubschman J.P., Heilwell G., Franco-Cardenas V., Pan C.K., Ostrick R.M., Mickunas E., Gay R., Klimanskaya I., Lanza R. (2012). Embryonic stem cell trials for macular degeneration: A preliminary report. Lancet.

[91] Kauper K., McGovern C., Sherman S., Heatherton P., Rapoza R., Stabila P., Dean B., Lee A., Borges S., Bouchard B. (2012). Two-year intraocular delivery of ciliary neurotrophic factor by encapsulated cell technology implants in patients with chronic retinal degenerative diseases. Investig. Ophthalmol. Vis. Sci..

[92] Nadri S., Yazdani S., Arefian E., Gohari Z., Eslaminejad M.B., Kazemi B., Soleimani M. (2013). Mesenchymal stem cells from trabecular meshwork become photoreceptor-like cells on amniotic membrane. Neurosci. Lett..

[93] Siqueira R.C., Messias A., Voltarelli J.C., Messias K., Arcieri R.S., Jorge R. (2013). Resolution of macular oedema associated with retinitis pigmentosa after intravitreal use of autologous BM-derived hematopoietic stem cell transplantation. Bone Marrow Trans..

[94] Singh M.S., MacLaren R.E. (2011). Stem cells as a therapeutic tool for the blind: Biology and future prospects. Proc. Biol. Sci. R. Soc. B.

[95] Tao Y.X., Xu H.W., Yin Z.Q., FitzGibbon T. (2010). Noggin induces human bone marrow-derived mesenchymal stem cells to differentiate into neural and photoreceptor cells. Indian J. Exp. Biol..

[96] Nadri S., Kazemi B., Eeslaminejad M.B., Yazdani S., Soleimani M. (2013). High yield of cells committed to the photoreceptor-like cells from conjunctiva mesenchymal stem cells on nanofibrous scaffolds. Mol. Biol. Rep..

[97] Tomita M., Adachi Y., Yamada H., Takahashi K., Kiuchi K., Oyaizu H., Ikebukuro K., Kaneda H., Matsumura M., Ikehara S. (2002). Bone marrow-derived stem cells can differentiate into retinal cells in injured rat retina. Stem Cells.

[98] Gong L.H., Wu Q., Song B.W., Lu B., Zhang Y. (2008). Differentiation of rat mesenchymal stem cells transplanted into the subretinal space of sodium iodate-injected rats. Clin. Exp. Ophthalmol..

[99] Arnhold S., Absenger Y., Klein H., Addicks K., Schraermeyer U. (2007). Transplantation of bone marrow-derived mesenchymal stem cells rescue photoreceptor cells in the dystrophic retina of the rhodopsin knockout mouse. Graefes Arch Clin. Exp..

[100] Hue D.M., Dong F.T., Yu W.H., Gao F. (2010). Differentiation of mesenchymal stem cell in the microenviroment of retinitis pigmentosa. Int. J. Ophthalmol.-Chi.

[101] Colozza G., Locker M., Perron M. (2012). Shaping the eye from embryonic stem cells: Biological and medical implications. World J. Stem Cells.

[102] Wang S., Lu B., Girman S., Duan J., McFarland T., Zhang Q.S., Grompe M., Adamus G., Appukuttan B., Lund R. (2010). Non-invasive stem cell therapy in a rat model for retinal degeneration and vascular pathology. PLoS One.

[103] Mauri M., Lentini D., Gravati M., Foudah D., Biella G., Costa B., Toselli M., Parenti M., Coco S. (2012). Mesenchymal stem cells enhance GABAergic transmission in co-cultured hippocampal neurons. Mol. Cell. Neurosci..

[104] Smith L.E. (2004). Bone marrow-derived stem cells preserve cone vision in retinitis pigmentosa. J. Clin. Investig..

[105] Otani A., Dorrell M.I., Kinder K., Moreno S.K., Nusinowitz S., Banin E., Heckenlively J., Friedlander M. (2004). Rescue of retinal degeneration by intravitreally injected adult bone marrow-derived lineage-negative hematopoietic stem cells. J. Clin. Investig..

[106] Bartsch U., Oriyakhel W., Kenna P.F., Linke S., Richard G., Petrowitz B., Humphries P., Farrar G.J., Ader M. (2008). Retinal cells integrate into the outer nuclear layer and differentiate into mature photoreceptors after subretinal transplantation into adult mice. Exp. Eye Res..

[107] Nei M. (1978). Estimation of average heterozygosity and genetic distance from a small number of individuals. Genetics.

[108] Selander R.K., Kaufman D.W. (1973). Genic variability and strategies of adaptation in animals. Proc. Natl. Acad. Sci. USA.

[109] Zhang X., Bok D. (1998). Transplantation of retinal pigment epithelial cells and immune response in the subretinal space. Investig. Ophthalmol. Vis. Sci..

[110] Hambright D., Park K.Y., Brooks M., McKay R., Swaroop A., Nasonkin I.O. (2012). Long-term survival and differentiation of retinal neurons derived from human embryonic stem cell lines in un-immunosuppressed mouse retina. Mol. Vis..

[111] MacLaren R.E., Buch P.K., Smith A.J., Balaggan K.S., MacNeil A., Taylor J.S., Osborne N.N., Ali R.R. (2006). *CNTF* gene transfer protects ganglion cells in rat retinae undergoing focal injury and branch vessel occlusion. Exp. Eye Res..

[112] Chen L.F., FitzGibbon T., He J.R., Yin Z.Q. (2012). Localization and developmental expression patterns of CSPG-cs56 (aggrecan) in normal and dystrophic retinas in two rat strains. Exp. Neurol..

[113] Tucker B.A., Park I.H., Qi S.D., Klassen H.J., Jiang C., Yao J., Redenti S., Daley G.Q., Young M.J. (2011). Transplantation of adult mouse iPS cell-derived photoreceptor precursors restores retinal structure and function in degenerative mice. PLoS One.

[114] Pearson R.A., Barber A.C., West E.L., MacLaren R.E., Duran Y., Bainbridge J.W., Sowden J.C., Ali R.R. (2010). Targeted disruption of outer limiting membrane junctional proteins (Crb1 and ZO-1) increases integration of transplanted photoreceptor precursors into the adult wild-type and degenerating retina. Cell Transpl..

[115] Jiang C., Klassen H., Zhang X., Young M. (2010). Laser injury promotes migration and integration of retinal progenitor cells into host retina. Mol. Vis..

[116] West E.L., Pearson R.A., Tschernutter M., Sowden J.C., MacLaren R.E., Ali R.R. (2008). Pharmacological disruption of the outer limiting membrane leads to increased retinal integration of transplanted photoreceptor precursors. Exp. Eye Res..

